# Neuroma of a double gallbladder: a case report

**DOI:** 10.1186/1757-1626-2-11

**Published:** 2009-01-06

**Authors:** Valentina Lefemine, Taha R Lazim

**Affiliations:** 1 All Wales Higher Surgical Training Programme, Glan Clwyd Hospital, Rhyl, LL18 5UJ, UK; 2 Royal Gwent Hospital, Newport, NP20 2UB, UK

## Abstract

We report a case of 55 year old male patient who presented with recurrent upper abdominal pain following a laparoscopic cholecystectomy. A subsequent diagnostic laparoscopy revealed the presence of a second gallbladder which was initially missed. The peculiarity of his symptoms can in part be explained by the presence of a traumatic neuroma in his second gallbladder. A subsequent cholecystectomy led to a complete resolution of this patient's signs and symptoms. As far as we know this is the first report in the literature of a traumatic neuroma in a second gallbladder.

## Introduction

The gallbladder is one of the organs in our body most subject to anatomical variations, its abnormalities may be related to number, shape and position and may also affect the cystic duct and cystic artery. Double and triple gallbladders are rare congenital anomalies with an incidence for the double gallbladder of 1: 3800–4000 patients [1]. All reports appeared in the literature are emphasising the importance of pre operative diagnosis, this should be achievable with the diagnostic tools available nowadays to the health practitioners. Diagnosis can easily be missed intraoperatively, especially laparoscopically, without prior knowledge of the abnormality.

We report a case of double gallbladder in a fifty five year old male patient in whom the two gallbladders were diagnosed in two separate occasions. The first one was symptomatic and removed laparoscopically, a second gallbladder was found a year later. Post cholecystectomy, his clinical presentation was not characteristic of biliary symptoms and signs and this led to delay in the diagnosis and management of the second gallbladder. The postoperative histological finding of a neuroma of the second gallbladder can in part explain the atypical nature of our patient's symptoms.

As far as we know this is the first report in the literature of a traumatic neuroma in a second gallbladder.

## Case presentation

A 55 years old man presented as an emergency with a week history of worsening right hypocondrium pain in keeping with a biliary colic pain. He was also complaining of anorexia and some weight loss.

His past medical history included hypertension, asthma, symptomatic hiatus hernia and diverticular disease. On his routine blood test, including full blood count, CRP, amylase and urea & electrolytes and liver function test, the only abnormal value was the bilirubin level which was raised at 44 umol/l.

An ultrasound scan of his abdomen demonstrated a small amount of echogenic sludge within the gallbladder but no calculi. The gallbladder wall was not thickened and there was no evidence of intra or extra hepatic biliary tree dilatation. The second gallbladder was not visualised, a this time, however the radiographer commented on the poor quality of the images due to overlying bowel gas. The patient was discharged home as he was in good clinical condition and brought back a month later for an elective laparoscopic cholecystectomy (not by the authors).

The intraoperative findings, as commented in the operative notes, were a distended gallbladder with some omental adhesion to its wall. The histology report confirmed the specimen to be a chronically mild inflamed gallbladder containing stones.

The patient had a quick and uneventful post operative recovery.

The same patient was readmitted to hospital eight times following his operation.

His symptoms represented few months later with recurrent attacks (every two to three weeks) of persistent, sharp pain in the right hypocondrium, periumbelical and epigastric region, very rarely radiated to the back. The pain lasted for about thirty minutes and was exacerbated by movement such as bending and stretching and in some occasions, but not always, was triggered by eating. However there was no significant association with eating fatty food. He was also experiencing a spasmodic type of pain of few seconds in duration, associated with severe nausea and occasional vomiting, this pain being different from the biliary type of pain experienced previously.

During each admission routine blood tests were performed (full blood count, amylase, urea & electrolytes, liver function test) and resulted within normal limits except from his ALT, whose value had been fluctuating from normal level up to 70 um/l; and his bilirubin which had only one high reading of 23 umol/l.

Clinical examination had repeatedly shown a soft abdomen, tender in the right upper quadrant with no signs of peritonism and negative Murphy's sign. The patient was pyrexial only in one occasion, with a CRP of 46.5 but persistently normal white cell count.

Extensive radiological investigations were carried out in attempt to find a cause for our patient's recurrent pain (table 1).

**Table 1:** Radiological examinations and reports, in chronological order

Type of scan	Result
ULTRASOUND OF ABDOMEN	Normal liver. No intrahepatic duct dilatation. CBD 5 mm. Note made of previous cholecystectomy. Pancreas not clearly visualised. Normal spleen and kidneys
OGD	exudative gastritis involving antrum, evidence of bile reflux HP – ve
BARIUM FOLLOW THROUGH	The small bowel has a normal mucosal pattern and there is no evidence of obstruction
HIDA SCAN	Normal examination
MRCP	There is a gallbladder shaped fluid collection in the gallbladder fossa. This looks for all the world like a slightly contracted gallbladder but given the history a small persistent collection is perhaps more likely. It does seem to communicate with the cystic duct stump. The biliary tree is not dilated and no stones or strictures are seen within it. The previous HIDA scan shows a similar collection of contrast in the gallbladder fossa
C.T. CHOLANGIOGRAM	Well opacified biliary tree. The structure in the gallbladder bed is clearly demonstrated to be a residual gallbladder. There are clips at the fundal end and there appears to have been a kind of hemi-cholecystectomy. The residual gallbladder is in continuity with the cystic duct and onwards to the biliary tree. I can't see any gallstones in the residual gallbladder or biliary tree and there is certainly no biliary dilatation. No convincing evidence of any collections in the gallbladder fossa

**Figure 1:**
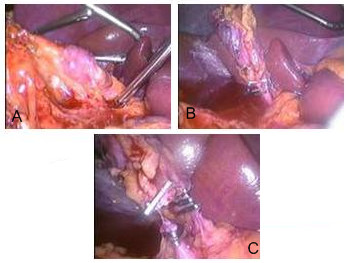
**a – Small, contracted second gallbladder lying on omentum**. Omental adhesions to the gallbladder released. **b **– Cystic duct of second gallbladder with three endoclips, the cystic duct is clearly demonstrated to enter the common bile duct. **c **– Cystic duct divided between endoclips and cystic artery clipped.

The HIDA scan was normal but the MRCP and CT cholangiogram raised the possibility of an incomplete cholecystectomy or a collection in the gallbladder bed communicating with the cystic duct stump. In view of the ambiguous radiological findings and persistent symptomatology a diagnostic laparoscopy was carried out, one year after his first laparoscopic cholecystectomy. The intraoperative findings were extensive intraabdominal adhesions and thick omental adhesions to the previous gallbladder bed; the second gallbladder was totally hidden to our own eyes, it was wrapped in omentum (figure 1a) and laying on the transverse colon. Its cystic duct (figure 1b) entered the common bile duct at lower level then usual and there were several small inflamed lymph nodes along its cystic artery (figure 1c).

Laparoscopic cholecystectomy was then performed by the authors. The histology report, apart from confirming the specimen to be a gallbladder, interestingly reported the presence of a traumatic neuroma within the gallbladder wall possibly relating to previous surgery. There was no evidence of dysplasia or malignancy. The patient was discharged home two days after the operation having made a full recovery.

He was reviewed in our outpatient clinic three weeks following his discharge from hospital and was finally pain free and in excellent clinical condition.

## Discussion

Double gallbladder is a rare congenital anomaly with an incidence of 1: 4000 patients [1]. However there are several reports in the literature of double and also triple gallbladder.

**Figure 2:**
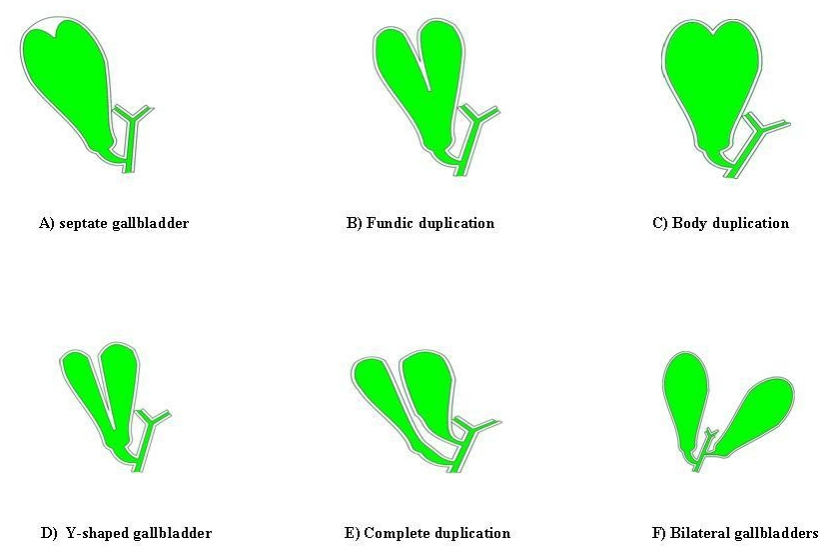
**Boyden's classification of gallbladder anatomical variations**.

Double gallbladders are classified according to the Boyden's classification[1] (Figure 2). The two main types of duplications are vesica fellea divisa (bilobed gallbladder) and, more commonly, vesica fellea duplex (true duplication), with two different cystic ducts. The true duplication is sub classified into H shaped type which entails two separate gallbladders and cystic ducts entering separately into the common bile duct; and Y shaped type, where the two cystic ducts unites before entering into the common bile duct. In our patient we found a complete duplication of the gallbladder with its own cystic artery and duct entering the common bile duct at lower level then normal.

Preoperative diagnosis of anatomical variations of the hepatobiliary tract is paramount in order to avoid potential damage to the duct system, besides abnormal anatomy may be easily overlooked during surgery[2] as it happened in this case.

Persisting signs and symptoms after cholecystectomy are generally referred to as postcholecystectomy syndrome, this generic entity entails a great variety of biliary tract pathologies [3] including rare ones such as neuromas of the biliary tract and pathological processes of an undiagnosed second gallbladder. Double gallbladders do not present with specific symptoms and the incidence of disease in this gallbladder is similar to its normal variant, with gallstone formation being the commonest pathological process occurring in one or both lobes.

Traumatic or amputation neuromas are known to arise at the stump of the cystic bile duct after cholecystectomy, but traumatic neuromas arising in the gallbladder are extremely rare.

The first report in the literature of a traumatic neuroma of the gallbladder is dated back in 1985 by Sano et all. [4]. The authors described the case of a polypoid traumatic neuroma incidentally found in a gallbladder resected for cholelithiasis, the tumour had probably developed as the result of injury to the gallbladder during an unsuccessful cholecystectomy performed 20 years previously.

Matsuoka et all [5] in 1996 reported a case of traumatic neuroma of a gallbladder in the absence of surgery or cholelithiasis. Amputation neuromas of the biliary tract, especially of the common bile duct and cystic duct stump, following cholecystectomy have been described as early as 1946[6].

To our knowledge this is the first report of a traumatic neuroma of a duplex gallbladder. Most likely, during the first laparoscopic cholecystectomy the second gallbladder was accidentally injured as not identified at that time.

The gallbladder is extensively innervated by sympathetic and parasympathetic fibres (the latter running in the vagus nerve). Stimulation of the sympathetic component will produce mainly epigastric pain whilst vagal stimulation will cause dyspepsia and vomiting [7].

Our patient's main complain was upper abdominal pain which was atypical, not in keeping with a classic biliary colic or acute cholecystitis type of pain. In retrospect we think his pain or some elements of it might have been caused specifically by the neuroma arisen in the second gallbladder wall.

Neuromas are composed of sympathetic fibres whose compression or distension will produce a sharp, constant pain [8] which will be mostly localised in the epigastric region if the neuroma arises from the gallbladder. Compression or distension of the tumour in our case could have been caused by a distended colon or duodenum, which could likely explain why our patient reported, in some occasion, relief of the pain after bowel openings; adhesions, which were extensive in this case, are also recognised cause of pain: by exerting traction on the gallbladder wall they will cause tension of the neuroma fibres; distension of the gallbladder will also cause stretching of the neuroma fibres eliciting pain.

We believe that the peculiarity of signs and symptoms and the persistent negative radiological investigations have been fundamental contributing factors to the delay of the diagnosis. Nevertheless we would like to highlight the importance of awareness of the possibility of a double gallbladder which could potentially be affected by any pathological processes commonly found in the hepatobilary tract. In case of persisting symptoms after laparoscopic cholecystectomy the possibility of the existence of a second gallbladder needs to be bore in mind as this can lead to diagnose this peculiar condition at an earlier stage. Even though modern radiological techniques can sometimes fail to spot such abnormalities without a high index of suspicion, the focus should be on preoperative diagnosis of these variations as they can be easily missed at the time of surgery leading to intraoperative complications, persistent symptoms after surgery and often the need of performing further surgical procedures.

## Consent

Written informed consent was obtained from the patient for publication of this case report and accompanying images. A copy of the written consent is available for review by the Editor-in-Chief of this journal.

## Competing interests

The authors declare that they have no competing interests.

## Authors' contributions

VL was a major contributor in writing the manuscript. All authors read and approved the final manuscript. TL performed the surgery and helped reviewing the manuscript.
